# Proceedings of the first African Health Forum: effective partnerships and intersectoral collaborations are critical for attainment of Universal Health Coverage in Africa

**DOI:** 10.1186/s12919-018-0104-2

**Published:** 2018-07-03

**Authors:** Martin Okechukwu C. Ota, Doris Gatwiri Kirigia, Emil Asamoah-Odei, Pamela Suzanne Drameh-Avognon, Olushayo Olu, Mwelecele Ntuli Malecela, Joseph Waogodo Cabore, Matshidiso Rebecca Moeti

**Affiliations:** 1 WHO Regional Office for Africa, Brazzaville, Congo; 2 Consultant, Regional Director’s Office, WHO Regional Office for Africa, Brazzaville, Congo; 3 WHO Country Office, Kigali, Rwanda

**Keywords:** First Africa health forum, Sustainable development goals, Universal health coverage, Partnerships, Intersectoral collaborations, Private sector, Research and innovative approaches, Africa

## Abstract

**Background:**

Universal Health Coverage (UHC)is central to the health Sustainable Development Goals(SDG). Working towards UHC is a powerful mechanism for achieving the right to health and promoting human development which is a priority area of focus for the World Health Organization WHO. As a result, the WHO Regional Office for Africa convened the first-ever Africa Health Forum, co- hosted by the government of Rwanda in Kigali in June 2017 with the theme *“Putting People First: The Road to Universal Health Coverage in Africa”.* The Forum aimed to strengthen and forge new partnerships, align priorities and galvanize commitment to advance the health agenda in Africa in order to attain UHC and the SDGs. This paper reports the proceedings and conclusions of the forum.

**Methods:**

The forum was attended by over 800 participants. It employed moderated panel and public discussions, and side events with political leaders, policy makers and technicians from ministries of health and finance, United Nations agencies, the private sector, the academia, philanthropic foundations, youth, women and non-governmental organizations drawn from within and outside the Region.

**Conclusions:**

The commitment to achieve UHC was a collective expression of the belief that all people should have access to the health services they need without risk of financial hardship. The attainment of UHC will require a significant paradigm shift, including development of new partnerships especially public-private partnerships in selected areas with limited government resources, intersectoral collaboration to engage in interventions that affect health but are outside the purview of the ministries in charge of health and identification of public health issues where knowledge gaps exist as research priorities. The deliberations of the Forum culminated into a *“Call-to-Action” – Putting People First: The Road to Universal Health Coverage in Africa,* which pledged a renewed determination for Member States, in partnership with the private Sector, WHO, other UN Agencies and partners to support the attainment of the SDGs and UHC. There was agreement that immediate action was required to implement the call-to-action, and that the African Regional Office of WHO should develop a plan to rapidly operationalize the outcomes of the meeting.

**Electronic supplementary material:**

The online version of this article (10.1186/s12919-018-0104-2) contains supplementary material, which is available to authorized users.

## Background

Over the past 25 years concerted efforts by African governments and partners to focus on the Millennium Development Goals (MDGs) led to substantial improvements in population health outcomes. This is reflected in better availability and utilization of health services targeted at addressing the priority disease burdens. These achievements are mainly associated with an increase in total health expenditure and general government expenditure on health. Despite the progress made, the health-related MDG targets were not achieved in most countries by the end of 2015 [1]. In many countries where some good progress was made, the benefits were only enjoyed by certain population groups. Currently, the burden of communicable diseases is still high and has been further complicated by the rising prevalence of non-communicable conditions resulting in a double burden of diseases. In addition, recurrent emerging public health emergencies continue to undermine health systems and communities, disrupt national economic activities and threaten peace and security on the continent. These events occur against the backdrop of a demographic shift, rapid urbanization and climate change. These factors constitute determinants of health, which reside outside the control of ministries in charge of health yet have huge effects on health and require intersectoral actions.

In 2015, Heads of State and Governments of UN Member States agreed on the Sustainable Development Goals (SDGs) that articulates the need for countries to ensure healthy lives and promote well-being for all at all ages [2]. Central to the health SDG is the Universal Health Coverage (UHC), the eighth target in Goal 3, which is defined as “all people receiving the quality promotive, preventive, curative, rehabilitative and palliative services they need without suffering financial hardship in so doing” [3]. This target underpins the achievement of all the other health and related SDG targets. Working towards UHC is a powerful mechanism for achieving the right to health and promoting human development, and is a priority area of focus of the World Health Organization’s (WHO) thirteenth General Programme of Work that aims to achieve “triple billion” more people benefitting from UHC, protected from health emergencies, and enjoying better health and well-being.

The SDGs are ambitious, and their attainment, particularly for the African Region, will require a significant paradigm shift including leveraging innovative approaches. One of the challenges of the MDGs was the limited focus on interventions that affect health but reside outside the purview of the ministries in charge of health, such as those targeting the social, economic and environmental determinants of health. This requires strengthened partnerships and intersectoral collaboration as the cornerstones for the attainment of UHC and the SDGs. In response to the foregoing and in keeping with the Transformation Agenda [4, 5], a strategy of the WHO Regional Director for Africa to accelerate implementation of the WHO reform within the African Region, the first-ever WHO Africa Health Forum was convened. It was hosted by the Government of the Republic of Rwanda, from 27 to 28 June 2017 in Kigali. The theme of the Forum was *Putting People First: The Road to Universal Health Coverage in Africa.* The aim of the Forum was to strengthen and forge new partnerships, align priorities and galvanize commitment from national political leaders, civil society and the private sector in advancing the health agenda in Africa, and attaining the SDGs. This paper summarizes the proceedings of the Forum and concludes on what is needed to implement its call-to-action and the attainment of SDGs and UHC in the African Region.

## Methods

The forum employed moderated plenary discussions, side and special events with political leaders, health experts, stakeholders and partners drawn from within and outside the Region; an additional file shows the details of the sessions and speakers [see Additional file 1]. Deliberations took place in a total of six sessions of panel discussions, two side events and one special event in which the discussants offered their expert opinions on the way forward on key thematic areas for the attainment of the SDGs and UHC. The side and special events provided opportunities to further drill down on important issues and innovative ideas such as digital health, youths and adolescents that are critical to the attainment of the UHC and SDGs. The Forum was attended by over 800 participants representing a broad spectrum of the society, ranging from political leaders and policy makers, ministries of health and finance, development partners, inter-governmental agencies, other United Nations agencies, and Regional Economic Communities from Africa and the rest of the World. The private sector, academia, philanthropic foundations, youth and women organizations, non-governmental organizations, civil society organizations and the media were also well represented at the forum. It ended with a call-to-action which is based on the main conclusions and recommendations from the different sessions. Dedicated rapporteurs documented the key highlights of all sessions and events. The authors who include some of the dedicated rapporteurs extracted and reviewed the relevant sections of the discussions of the Forum to synthesize these proceedings.

### Proceedings

#### Panel discussions

##### Session 1: Health financing: getting value for money - taking financial responsibility for our own health

In 2001, African Member States of WHO committed to the Abuja Target [6] of increasing their health budgets to 15% of government expenditure and in 2015 they endorsed a Declaration on sustainable financing for Development in Addis Ababa, Ethiopia [7]. Despite the commitments, domestic investment for health in most WHO African countries falls short of the 15% mark for public financing. As a consequence, out of pocket expenditure remains high with 11 million persons impoverished due to catastrophic medical expenses annually [8]. The increase in Total Health Expenditure (THE) per capita from 40 USD in 1995 to 109 USD in 2013 in the Region was due to an estimated annual increase of 11.3% in external financing from Development Assistance for Health (DAH) [9]. This growth has since slowed to 4.3% between 2010 and 2015, a proof that funding from external sources is unsustainable. Therefore, countries have to innovate in how they can efficiently utilize the available limited resources to provide access to essential health care services while exploring mechanisms to increase domestic sources of revenue to finance UHC. Sustainable health financing derived from domestic sources has long been recognized as an important health system input for the achievement of UHC and the SDGs. As a result the region has spawned numerous health reforms related to health financing, health insurance and results or performance based financing among others. These have been influential in redefining how finances for health are mobilized, pooled, and used to provide access to quality essential health care services.

It was stressed that investing in UHC is important to advance human capital development, which is critical for economic and social development and governments should therefore prioritize spending to ensure they achieve the SDGs in the remaining 5000 days to 2030. The guiding principle to ensuring action for sustainable financing for health is to recognize health as a human right, and governments are required to take greater responsibility by increasing domestic funding and not rely solely on external financing and out-of-pocket payments by households. To achieve this goal entails taking into consideration health in the national development agenda, exploring ways of instituting mandatory social protection and prepaid health insurance using innovative financing mechanisms such as earmarked taxes. It is also important for countries to reduce wastage arising from fragmentation by harmonizing health financing funds, and ensuring that mobilized resources are managed effectively. The private sector engagement for health innovative digital financing and health care service expansion can be leveraged to support UHC attainment. The responsibility for health, health financing, good leadership and governance to improve health gains is everyone’s business. The session concluded that there is a need for more evidence-based advocacy for increasing and sustaining health financing with political leaders, private sector, civil society, local faith-based organizations to ensure that financing for health is prioritized. Similarly, the citizens in turn must be empowered to recognize and demand for this right to health and ensure that they are represented at decision-making levels to facilitate evidence-informed demand for better health.

##### Session 2: Global health security: protecting the people of Africa

The African Region deals with more health emergencies than any other region. Each year there are over 100 outbreaks and other health emergencies, resulting in unacceptably high morbidity, mortality, disability and socioeconomic disruptions including weakening of already fragile health systems. Despite the availability of existing frameworks and strategies such as: the International Health Regulations (IHR, 2005); the WHO African Region’s integrated disease surveillance and response (IDSR) strategy that hinges on an “all-hazards approach”, and the African health disaster risk management (DRM) strategy [10], tackling outbreaks and other health emergencies continues to be a challenge. This is largely due to the fragmented implementation of preparedness and response interventions, limited intersectoral cooperation and coordination, inadequate resources, weak health systems, and inadequate IHR (2005) core capacities in countries.

Participants at this session highlighted that apart from the associated high morbidity and mortality, the recurrent outbreaks also threaten national, regional and global health security. This underscores the need to invest in stronger emergency preparedness, risk reduction, response and recovery through holistic health disaster risk management and active engagement of communities as a more cost effective alternative to controlling epidemics. Developing capacities for early detection, rapid response and prompt recovery is essential, as well as strengthening health systems. The community should be strongly involved and integrated in the health security strengthening. This may entail involvement of strategic partners and stakeholders, within and beyond the health sector, and mobilization of better global advocacy. An approach could be the use of existing platforms such as the International Federation of Red Cross and Red Crescent Societies with a network of about 1.8 million volunteers in 49 countries. It utilizes a community-centred and integrated framework to address key issues such as livelihood, food security and disaster risk management. The social, economic and environmental determinants that influence vulnerability, risk and outcomes related to health emergencies should also be addressed using similar frameworks.

The case of Liberia’s experience in tackling the Ebola epidemic is a lesson that stakeholders and partners must learn to work intersectorally and support each other to facilitate the development of strong, resilient and inclusive health systems. Such a health system involves sensitive early warning systems, contributions from informed, empowered and engaged communities including strong laboratory and logistics capacities and infrastructure. Skilled and motivated health workforce is critical to ensure delivery of safe, effective, quality health care services including accessibility to appropriate medical counter-measures, vaccines and drugs.

##### Session 3: Health research, innovation and data for sustainable development

The World Health Assembly endorsed that policy and decision making for sustainable development should be based on evidence from research and routine health data [11]. However, the performance of health information systems in the WHO African Region is sub-optimal. For instance, coverage of civil registration and vital statistics in most countries is very low, with coverage in most countries less than 50%. There are major challenges with population denominator data as projections from the Central Statistics Office are often inaccurate. In addition, the availability of disaggregated (including socioeconomic status, education, sex, and urban/rural residence) and quality data is low. Inequalities are multidimensional and monitoring the extent of inequality may vary considerably across different dimensions such as economic status, education, sex, and urban/rural residence which are fundamental to the equitable and progressive realization of UHC and SDGs [12].

Research for health generates timely and reliable health information to facilitate improvement of equitable distribution of high quality health services including discovering of new tools to improve people’s health and accelerate the rate of global, regional, and national development. The region with such huge health challenges paradoxically has not given priority to health research, which underlines the current weakness of the health research systems [13, 14]. The research arena in the African Region is characterized by a multiplicity of actors, externally driven agenda, dispersed efforts and unclear results in relation to impact on country specific priority health problems. There is weak research capacity in most of the countries as a result of inability to train and retain researchers. There is also poor research coordination, failure to harmonize and align donors and partners around country research strategies and priorities, which contribute to duplication of efforts, waste of resources and inability to detect and track research and innovations. These challenges will hinder the ability of the Region to generate and use evidence to guide its efforts towards achievement of the SDGs and UHC if left unmitigated.

Some of the major themes highlighted in the discussion include the critical need to generate local knowledge for evidence-based decision making. The availability of disaggregated, quality and timely data was considered as the centerpiece for efficient and effective monitoring of progress and performance towards the attainment of the SDGs and for facilitating decision making. The discussants emphasized the need to develop high-quality health information systems that are linked to review and evaluation cycles across health systems in all countries in the African region. The stakeholders’ consensus was that these actions will support equity-oriented progressive realization of evidence-based UHC and SDGs.

The session concluded that countries should strengthen efforts for the collection of health data including research, civil registration and vital statistics, web-based facility reporting, regular household and facility assessment surveys, regular reviews of quality of routine health facility data, as well as harmonization and alignment of data reporting requirements of donors and partners. Importantly, countries should adopt an open data-sharing policy and develop a supportive legal and administrative framework to facilitate sharing and use of data in accordance with agreed standards for confidentiality and data security. Importantly, there is need for each country to identify public health issues where knowledge gaps exist as research priorities. In particular, there should be emphasis on identifying ways to increase the coverage of existing tools for improving the health of the population (implementation research), share lessons learnt as well as discover new interventions. Countries should establish sustainable financing mechanisms to strengthen the functions of their research systems, in particular, the strengthening of infrastructure and the human resources to address the identified research agenda, improve uptake of the research products and reduce the ‘brain drain’ from the Region [15]. Moreover, South-South as well as North-South collaborations should be encouraged to share knowledge and strengthen research capacity. Government support and ownership is critical in implementing and creating conducive environment for the optimization of the conduct and use of research.

##### Session 4: Making UHC work in Africa – potential private sector contributions

The SDGs present an unprecedented opportunity and a framework for collaboration across all sectors, including the private sector, for its achievement. The private sector is described as, “comprising all providers who exist outside the public sector, whether their aim is for philanthropic or commercial purposes” [16, 17]. UHC particularly requires a strengthened, responsive and resilient health system which can be achieved with support from the private sector. Engagement of the private sector should ideally maximize strategic areas such as efficiency, innovation, capacity development, expansion of health care services coverage, health financing and equity. This will allow governments and ministries in charge of health to focus on advancing expertise, their regulatory functions and other priority areas [18].

At the country, regional and global levels, public-private partnership can facilitate common interest in building healthier societies by advocating for universal health coverage. The development of the conjugate meningococcal meningitis A vaccine (MenAfriVac) through the Meningitis Vaccine Project is a good example of how public-private partnership can be successfully used to advance the UHC agenda. This was a collaborative venture involving WHO, PATH, the Serum Institute of India Private Ltd., and public health officials across Africa to develop an affordable, tailor-made and effective vaccine for use against meningitis A in sub-Saharan Africa [19].

The session concluded that opportunities and modalities of engagement between the public and private sector can take many different forms. This includes various levels of engagement, commitment, and financial risk-sharing, ranging from technical assistance, capacity development, consulting, to outsourcing. Other areas are corporate social responsibility actions, financing, public-private partnership projects, advocacy, innovations, and local markets and manufacturing. A paradigm shift from the government’s standpoint is fundamental to optimally engage the private sector in recognition that the government cannot do it alone. It is also critical to build trust between the public and private sectors. Policy makers have to open the door for collaboration with the private sector and partners to create innovative solutions to health system challenges. Governments should therefore put-in-place regulatory frameworks and accreditation systems that will address different needs of the private sector to aid collaboration and partnerships.

##### Session 5: New public health threats (non-communicable diseases; urbanization; climate change)

While the continent made good progress in the prevention and control of “old enemies” such as malaria, tuberculosis and HIV/AIDS during the MDG era, new threats such as non-communicable diseases (NCDs) and the impact of climate change are jeopardizing the human, social and economic development of African populations [20]. In 2015, NCDs contributed 28% of total Disability Adjusted Life Years (DALYs) in the African Region as compared to 18% in 2000. DALYs due to NCDs for the same period increased from 124 million to 174 million. NCDs were responsible for about 33% of all deaths recorded in the Africa Region [21]. It is estimated that by 2030, NCDs will be responsible for 5 million deaths in the Africa Region [22]. In addition, climate change is worsening environmental conditions that exacerbate health vulnerabilities that are common in Africa and causes both direct and indirect impact on human health and well-being. It is estimated that 23% of premature deaths in the Region are attributable to unhealthy environments [23].

This session emphasized the need to put in place a comprehensive, evidence-based and coordinated response to the unfinished business of communicable diseases, tackle the high burden of NCDs, and address climate change and environmental determinants of health in the African Region. The historical attitude of vertical approaches must change through implementation of holistic patients-centred methods of better care and as opposed to fragmented and vertical disease prevention strategies. There is a need to incorporate NCDs demographic and service availability mapping surveys to inform service provision. This will also facilitate capacity building of health care providers of all cadres and players at all health system levels. Palliative care for NCDs and other chronic conditions, including HIV, should be developed as part of comprehensive care responses.

Climate change and social determinants of health pose serious threats to public health [24] and should be addressed concurrently through strategic and decisive approaches to ensure the attainment of UHC. This will require multifaceted approach and well-coordinated intersectoral actions to ensure that the required contributions from other sectors are addressed. Necessary regulatory frameworks to promote good practices and climatic and sociocultural behaviors are required to enhance health and well-being. Measures to control proliferation of disease risk factors should be put in place as part of national responses to old and emerging diseases and other threats to health. For example, the regulation to control dumping of wastes, advertisement and consumption of tobacco and tobacco products, sugars and oils. Decentralization of health services delivery with enforcement of minimum standards and packages will also be important to ensure delivery of quality services to all citizens at all levels.

##### Session 6: Putting people first – bringing better health to Africa’s people

The concluding session drew on the key issues and conclusions raised in the previous five sessions to strategize on how to bring better health to the people of Africa. Better health for all Africa’s people implies that everyone has access to all the health services they need in all settings without any financial hardship. People should demand equality of citizenship and should want, and have a right to expect, quality services with dignity and respect. Therefore, UHC would be expected to take a holistic approach that considers the different needs along the lifecycle and reducing the historical health inequities. Addressing the existing health inequities entails improving the scope and mode of delivery of health services that are critical for the attainment of the SDGs and UHC. To achieve this, the health sector interventions will need to be complemented by inter-sectoral action to effectively address the social, economic and environmental determinants of health [25]. Countries should identify ways to optimize the contributions from a multiplicity of stakeholders and the different resources to build a resilient health system that will sustainably address the health and well-being of all the people without leaving anyone behind [2]. Empowerment of different stakeholders will also be critical and in particular the communities so they can participate and make informed decisions for their health [26]. Effective partnership calls for clear definitions of roles and responsibilities as well as for mutual accountability. Therefore deliberate efforts will be required to be put in place, and enforce the adherence to, enabling regulatory frameworks to ensure relevant gender and human rights to health. Strong public-private partnerships, effective inter-sectoral actions, adequate resource mobilization, allocation and use, and good governance systems will be necessary. Taken together, decentralization, primary health care approach, prioritizing and targeting the poor and the vulnerable population and addressing the other needs of the people are critical as we “put people first”. Government leadership and stewardship again are critical for effective coordination and partnership for health.

### Side and special events

#### Side event on using digital health services to achieve UHC and the SDGs in the African region

The African Region is experiencing a rapid development of information and communication technology (ICT), with 73.5% mobile and 20.7% internet user’s penetration, against 96% and 43% global levels. This side event discussed priorities and mechanisms for leveraging this rapid ICT development to scale up eHealth solutions towards strengthening health systems for the attainment of the SDGs and UHC. The discussions highlighted the timeliness to scale-up the eHealth solutions as mitigation to some of the health systems challenges such as shortage of human resources and weak infrastructure. In line with this, the WHO Regional Office for Africa has pursued partnerships and collaborations with potential organizations, and this session witnessed the launch of a joint partnership between WHO and the International Telecommunication Union (ITU) to leverage strategic opportunities for the adoption of ICT in the health sector.

### Special event on adolescent health, our flagship programme

The African continent is the only region in the world where the number of adolescents is predicted to increase over the next fifty years. The proportion of the world’s adolescent and youth living in Africa is expected to rise from 18% in 2012 to 28% by 2040, while the shares for all other regions will decline [27]. Within this context, improving the health and development of the African’s adolescents will be important to achieving the SDGs, and has become the main theme of the WHO African Region flagship programme. The objective of this special session was to elicit the engagement of Member States, partners and young people to contribute in improving the special health needs of adolescent population group. The keynote address for the event was delivered by the Special Guest of Honour, Her Excellency Mrs Jeanette Kagame who emphasized the need for more investments in adolescent health and to improve equity in delivering services for adolescents. She commended the WHO Adolescent Health Flagship Programme and called upon African countries to implement it and increase investments in the health and development of adolescents.

### Side event on engaging Africa’s youth towards achieving UHC

Africa is the continent with the world’s youngest population, with 70% of the region’s population being under the age of 30 years [28]. This can be viewed as Africa’s advantage over the rest of the world that has higher proportions of the older population. The WHO Regional Director for Africa remarked “The youth are Africa’s future leaders so we need to involve them at the stage of decision making in order to empower them to take responsibility for their health”. The youth should not just be beneficiaries of services, but they ought to be at the decision-making table and be involved in international forums, policy agendas as well as in the community. This should allow them to shape their own future and contribute to various policy decisions that affect them since they will bear the consequences for an extended period of time [29]. Governments were urged to make the education system strong enough by including leadership and policy matters, to empower and equip the youth to undertake these roles in the society.

### Call to action

The Forum culminated in a *“Call-to-Action” titled “Putting People First: The Road to Universal Health Coverage in Africa”* that highlights the public health needs for which the major stakeholders committed themselves to:

- Keeping UHC as the overarching approach for attaining SDG3 in order to ensure healthy lives and promote well-being for all at all ages;
- Sustaining strong political will and commitment, increasing and sustaining domestic and external financial contributions and investments in health, including establishing innovative financing mechanisms, ensuring value for money and increased accountability;
- Building, re-orienting and re-aligning health systems towards UHC, with emphasis on primary health care, and maintaining effective systems to ensure improved financial protection and affordability for the most vulnerable populations, including women, children and the youth while intensifying focus on quality and equity;
- Strengthening health workforce development and sustainability, including community health workers, to deliver quality health services;
- Empowering people, including the youth, with the information, skills and resources that will enable them to actively engage in health policy development and maintain healthy environments, improve health literacy thereby making effective decisions about their own health and that of their families and communities;
- Placing stronger focus on building national core capacities for the International Health Regulations, including outbreak and emergency preparedness and active engagement of communities, while mobilizing strategic partners within and beyond the health sector to address the social and environmental determinants that influence vulnerability related to health emergencies;
- Establishing well-coordinated multisectoral regional emergency mechanisms and teams to support countries for prompt response to outbreaks and other health emergencies supplementing national capacities when needed;
- Strengthening advocacy and national capacity for health research, including setting the agenda, improving infrastructure, regulatory mechanisms and human capacity for the generation, analysis, synthesis and use of research and other health data, and mobilizing the required funding;
- Promoting, through partnerships, the use of new technologies, including innovative eHealth solutions to support the attainment of UHC;
- Establishing well-coordinated multisectoral monitoring and progress-tracking mechanisms to promote efficiency and accountability in delivering on key health-related commitments to achieve concrete results towards the attainment of UHC;
- Creating new opportunities for improved partnerships and an enabling environment that brings together the different stakeholders to undertake transformational change, including strengthening legislative frameworks, regulatory capacity and financial management, and reorienting public policy-making and the health workforce.

## Conclusion

A number of key conclusions emanated from the Forum. First, the attainment of UHC will require a significant paradigm shift, including partnerships and intersectoral collaboration to engage in interventions that affect health but are outside the purview of the ministries in charge of health. Second, private sector engagement in selected areas with limited government resources is particularly important in low- and middle-income countries, to maximize efficiency, innovation and expand coverage to achieve national health goals. Third, the participants underscored the importance of the Forum and requested that similar meetings should be held biannually to further strengthen stakeholders’ engagement, identify common strategies and review progress towards the UHC and SDGs. Fourth, there was agreement that immediate action was required to implement the consensus reached, and that WHO in the African Region should develop an action plan to rapidly operationalize the outcomes of the Forum.

## Additional file

Additional file 1: Annotated programme of the first Africa Health Forum. (PDF 690 kb)

## Abbreviations

DAH: Development assistance for health
DALYs: Disability adjusted life years
DRM: Disaster risk management
ICT: Information and communication technology
IDSR: Integrated diseases surveillance and response
IHR: International health regulations
ITU: International Telecommunication Union
MDGs: Millennium development goals
NCDs: Noncommunicable diseases
SDGs: Sustainable development goals
THE: Total health expenditure
UHC: Universal health coverage
UN: United Nations
WHO: World Health Organization
