# Supplementary material for: Proceedings of the first African Health Forum: effective partnerships and intersectoral collaborations are critical for attainment of Universal Health Coverage in Africa
Source: BMC Proc. 2018 Jul 3;12(Suppl 7):8. doi: 10.1186/s12919-018-0104-2 (PMC6031170; doi:10.1186/s12919-018-0104-2)
Supplement: Supplementary file 1 — Annotated programme of the first Africa Health Forum. (PDF 690 kb) [file 12919_2018_104_MOESM1_ESM.pdf]

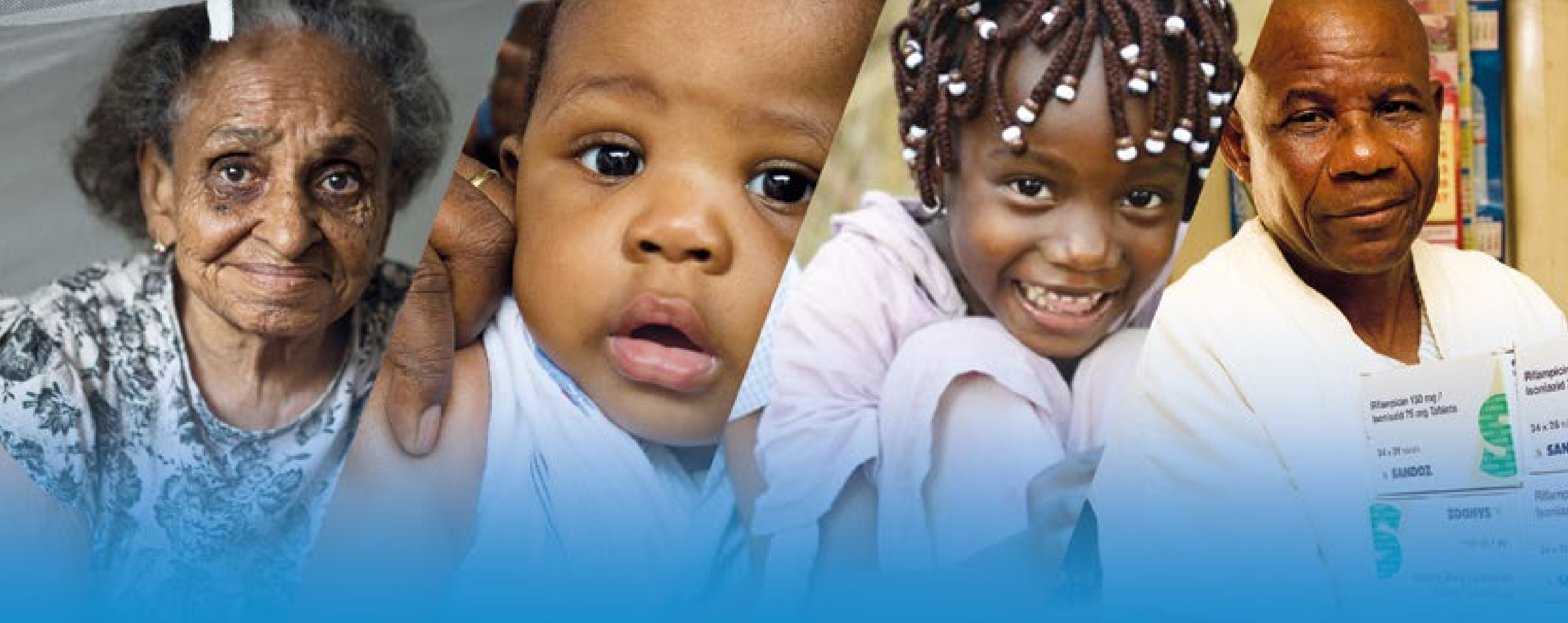

The First WHO
Africa Health Forum

PUTTING PEOPLE FIRST
The Road to Universal Health Coverage in Africa

Kigali, Rwanda, 27 – 28 June 2017

Republic of Rwanda

Ministry of Health

World Health Organization
REGIONAL OFFICE FOR Africa

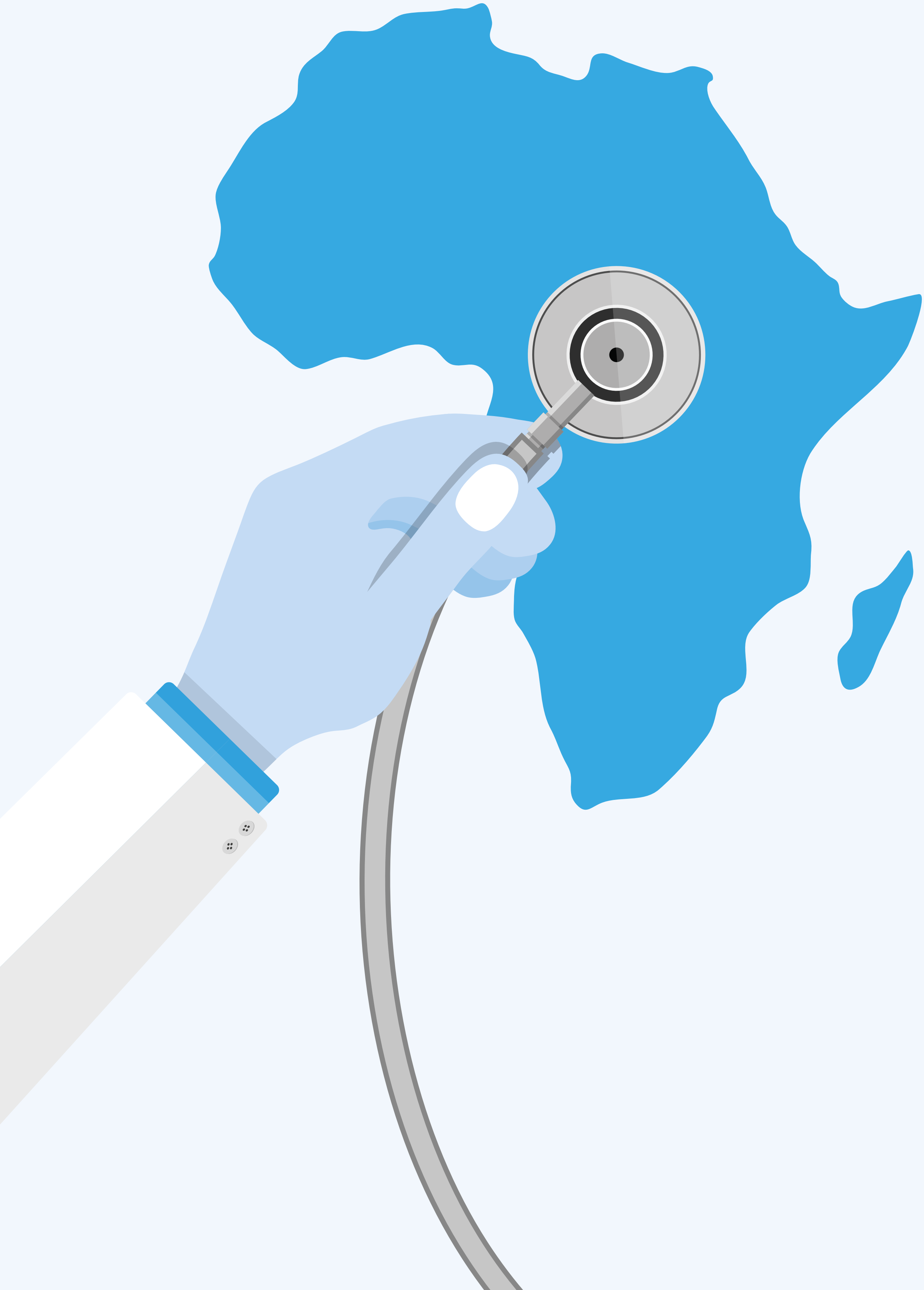

WELCOME

Welcome to the inaugural Africa Health Forum of WHO in the African Region. “Putting People First: The Road to Universal Health Coverage” is the Forum to discuss the future of healthcare across the continent, and subsequently the prosperity and economic welfare of our future.

I thank the Government of Rwanda for the kind hospitality and excellent arrangements made for this landmark event.

I am delighted you have joined us as strategic partners at this Forum. We all know that health is central to development and that partnerships are key to improving access to health and wellbeing. The Sustainable Development Goals reinforce the fact that a multisectoral approach is the foundation to Universal Health Coverage.

The Africa Health Forum brings together people from a wide spectrum of sectors to discuss challenges and opportunities for effective health service delivery and policy priorities in the Region. Furthermore, in line with our reform agenda, we are engaging in strategic partnerships with stakeholders particularly the youth, to strengthen their role in the future of health service delivery in Africa. We are putting people first to ensure that no one is left behind.

WHO in the African Region is committed to working with its Member States and partners to attain the highest possible level of health for Africa’s people by achieving the Sustainable Development Goals and in particular Universal Health Coverage for all.

I trust that you will find this Forum engaging, and that it will be the beginning of a fruitful partnership. Enjoy your stay in Kigali and we look forward to your continuous engagement and collaboration.

Yours sincerely

Dr Matshidiso Moeti
WHO Regional Director for Africa

ABOUT WHO AFRO

The goal of the WHO is to build a better, healthier future for people all over the world. WHO AFRO is one of the six regional offices of the organisation. As the technical agency and custodian of public health in Africa, the regional office seeks to galvanise political commitment, foster collaboration and align strategic priorities in advancing the health agenda in the region, especially around the Sustainable Development Goals. Strategic partnerships, effective engagement and coordinated action are critical for addressing the urgent and ever changing health needs of people living in Africa.

WHO AFRO SENIOR MANAGEMENT

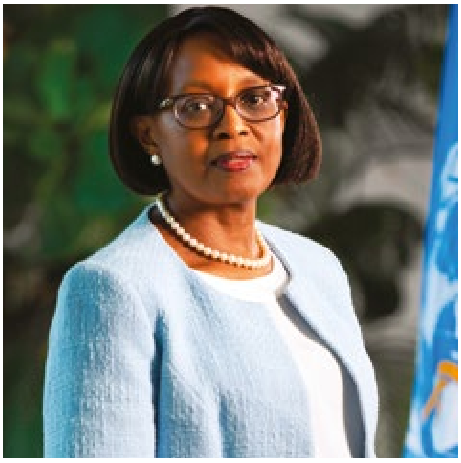

Dr Matshidiso Moeti
Regional Director for Africa

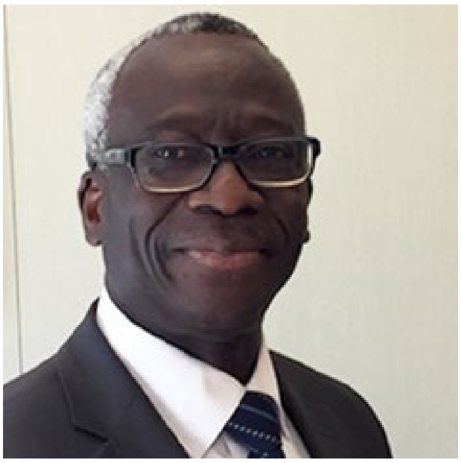

Dr Joseph Caboré
Director for Programme Management

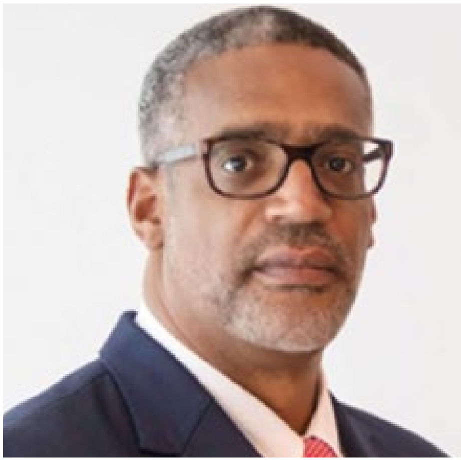

Mr Raul Thomas
Director, General Management

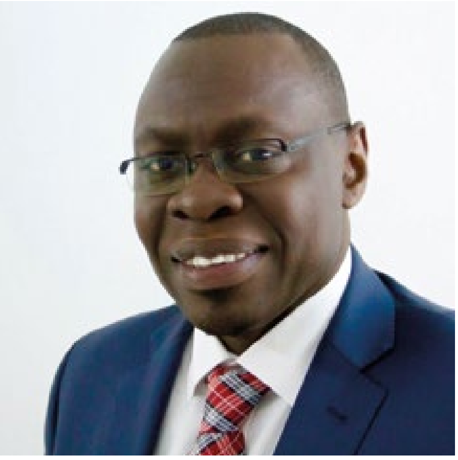

Dr Ibrahima Socé Fall
Director, Regional Emergencies

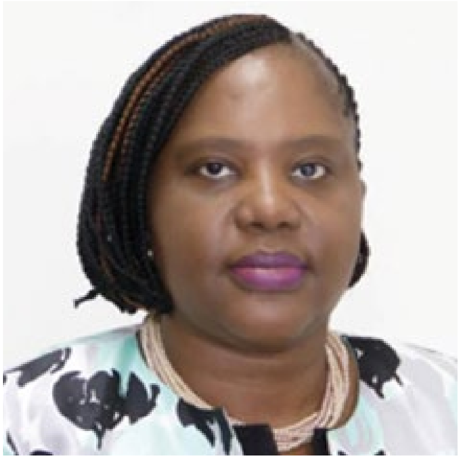

Dr Mwelecele Ntuli Malecela
Director, Office of Regional Director

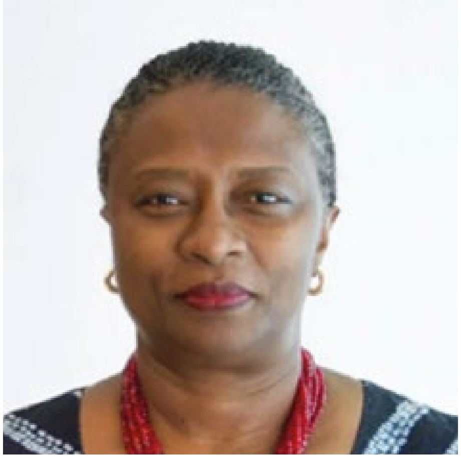

Dr Magda Robalo
Director, Communicable Diseases

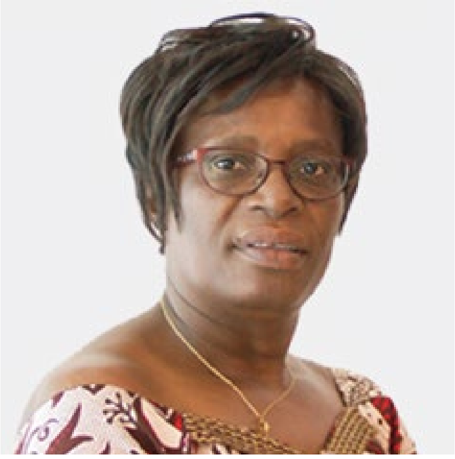

Dr Felicitas Zawaira
Director, Family and Reproduction Health

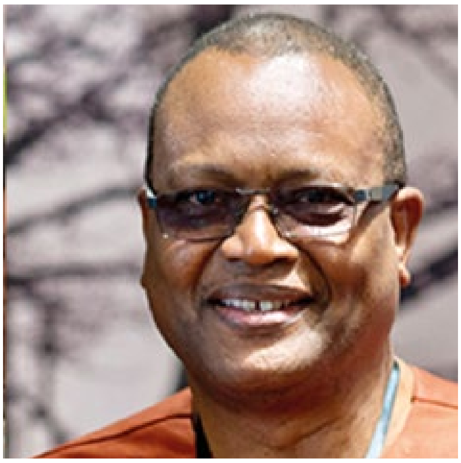

Dr Delanyo Dovlo
Director, Health Systems and Services

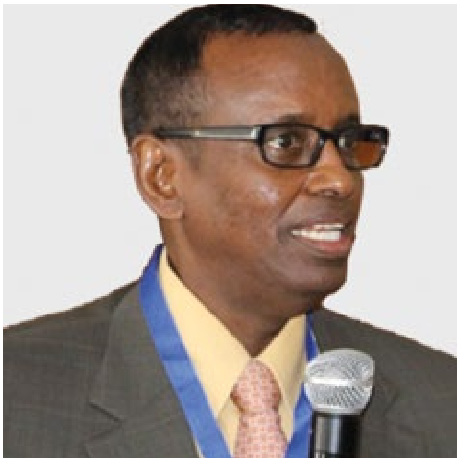

Dr Abdikamal Alisalad
Director, Non-communicable Diseases

FORUM PROGRAMME

DAY 1
27 JUNE 2017

09:00 am – 10:00 am
Registration – Kigali Heights: 2nd floor Eastern Wing opposite the Kigali Convention Centre

Networking – Foyer 1A at the Kigali Convention Centre

10:00 AM – 11:00 PM | Auditorium

Opening Ceremony

Master of Ceremony
Dr Jean Pierre NYEMAZI, Permanent secretary, Ministry of Health

Welcome remarks
Dr Matshidiso Moeti, WHO Regional Director for Africa

Keynote address
Guest of Honor

11:00 am – 11:30 am
Health break & networking – Foyer 1A

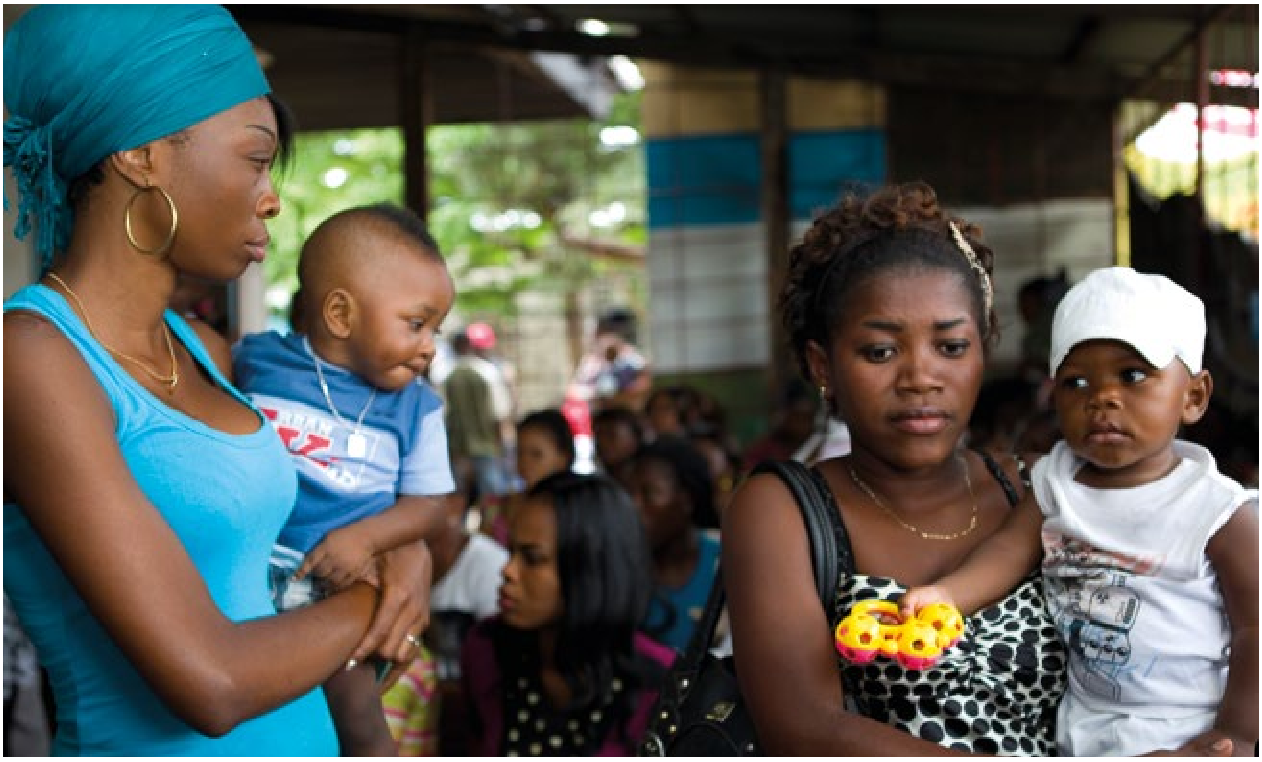

11:30 AM – 1:00 PM | Auditorium

Session 1 : Health financing: Getting value for money – Taking financial responsibility for our own health

Sustainable health financing has long been recognised as an essential part of Universal Health Coverage and an important plank in achieving the aims of the Sustainable Development Goals. In recent years the concept has led to numerous health reforms including health financing, health insurance and results-based financing reforms. These have been influential in redefining how finances for health are mobilised, managed and used to purchase essential health services across Africa.

Moderator

Mr Andrew Mwenda, Journalist & Founder, the Independent, Uganda

Keynote speaker

Dr Timothy G. Evans, Senior Director, The World Bank Group, USA

Panelists

Dr Uzziel Ndagijimana, Minister of State in Rwanda's Ministry of Finance

Mr Carl Manlan, COO, Ecobank Foundation, Ghana

Hon. Sarah Opendi, Minister of State for Health (General Duties) Uganda

Dr Githinji Gitahi, Group CEO, Amref Health Africa Headquarters, Kenya

Mr Fletcher Tembo, Director for the Making All Voices Count Programme, Kenya

1.00 PM – 2.00 PM
Lunch & networking – Foyer 1A

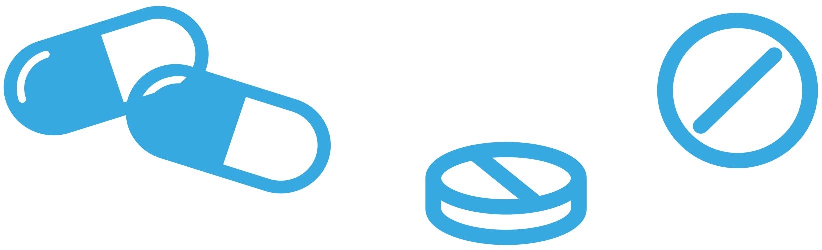

1:00 PM – 2:00 PM | AD10

Side event – E-Health

The session will witness the launch of a joint effort between WHO and the International Telecommunication Union (ITU) and the signing of a "Letter of Intent". ICT is already transforming how health care is delivered and how health systems are run. For ICT to achieve the health goals such as SDGs, and to ensure healthy lives and promote well-being for all for the African region, the proposed partnership will consolidate existing efforts and resources towards making available ICT foundations and platforms that are a requirement for providing and scaling up eHealth services. It will also focus on building a capable workforce to effectively use ICT. The partnership will address also the need of multi-stakeholders partnership models that can bring about sustainable adoption of Digital Health.

Moderator

Dr Delanyo Dovlo, Director, Health Systems and Services, WHO AFRO

Co – Chairs

Mr Andrew Rugege, ITU Regional Director for Africa

Dr Matshidiso Moeti, WHO Regional Director for Africa

Panelists

Hon. Prof. Léon Nzouba, Minister of Public Health and Population, Gabon

Hon. Prof. Isaac Folorunsho, Minister of Health, Nigeria

Hon. Dra. Nazira Karimo Vali Abdula, Minister of Health, Mozambique

Hon. Jean Philbert Nsengimana, Minister of Information Communication Technology, Rwanda

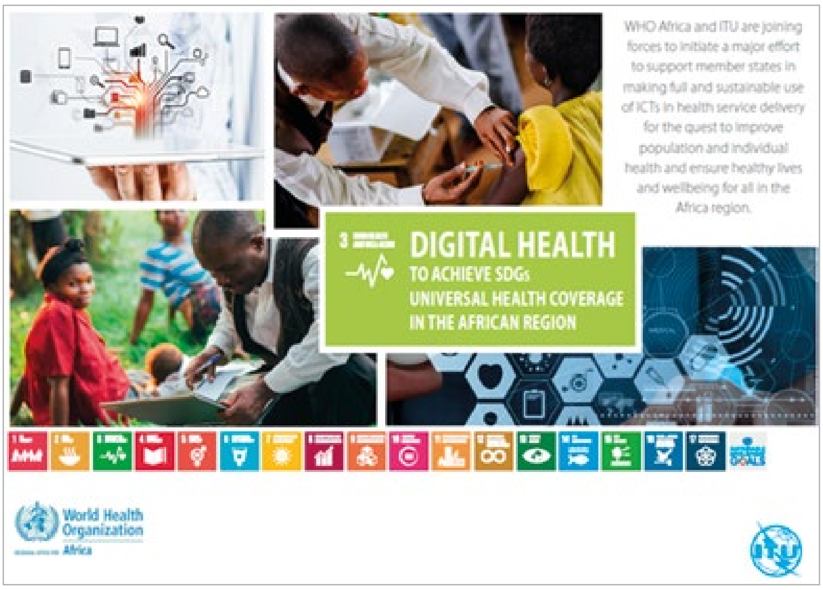

2:00 PM – 3:30 PM | Auditorium

Session 2: Global health security: Protecting the people of Africa

This panel will discuss lessons learned in managing various recent public health emergencies. It underscores the fact that public health emergencies have the potential to threaten African (indeed global) peace and security, disrupt national economic activities, and destroy health systems and communities.

Moderator

Ms Julie Gichuru, Founder & CEO, Arimus Media, Kenya

Keynote speaker

Dr Bernice Dahn, Minister of Health, Liberia

Panelists

Dr Donal Brown, Director East and Central Africa, Department for International Development, UK

Dr Adeiza Ben Adinoyi, Head of Africa Health and Care Unit, International Federation Red Cross, Kenya

Dr John Nkengasong, Director, Africa Centres for Disease Control and Prevention, Ethiopia

Dr Rebecca Martins, Director of the Centre of Global Health, US Centers for Disease Control and Prevention, USA

Mr Tewolde GebreMariam, CEO, Ethiopian Airlines, Ethiopia

Dr Emmanuel Ndahiro, Director, Rwanda Military Hospital, Rwanda

3:30 pm – 4:00 pm
Health break & networking – Foyer 1A

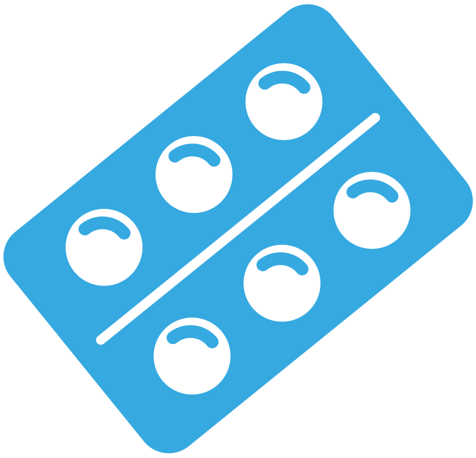

4:00 PM – 5:30 PM | Auditorium

Session 3: Health research, innovation and data for sustainable development

The African region has a high burden of diseases, with no interventions for many that disproportionally affect its population. This challenge will hinder achievement of the Sustainable Development Goals (SDGs) and Universal Health Care (UHC) if left unmitigated. The health research systems in the region could address this challenge if given the needed prominence. This session is aimed at advocating for a new era of health research and innovation to develop diagnostic, preventive and therapeutic interventions that will contribute to progress towards the SDGs and UHC.

Moderator

Mr Henry Bonsu, Journalist and Broadcaster, London, UK

Keynote speaker

Dr Michael Makanga, Executive Director, European & Developing Countries Clinical Trials Partnership (EDCTP), the Netherlands

Panelists

Dr Mary Amuyunzu-Nyamongo, Director and Technical Adviser, African Institute for Health and Development (AIHD), Kenya

Mr Joseph B. Babigumira, Assistant Professor, Global Health and Pharmacy, University of Washington, USA

Professor Pontiano Kaleebu, Director, Uganda Virus Research Institute (UVRI), Uganda

5:30 pm – 7:00 PM | AD10

Side event: Engaging Africa's Youth to achieve UHC

Moderator

Dr Waruguru Wanjau, Medical Doctor, Kenya

Opening remarks

Dr Matshidiso Moeti, Regional Director, WHO Regional Office for Africa, Congo

Panelists

Dr Githinji Gitahi, Group CEO, Amref Health Africa, Kenya

Dr Peter Okebukola, Associate Partner at McKinsey & Company, Nigeria

Mrs Abam Gladys Mambo-Doh, Director, Africa Ethics & Compliance Africa, GlaxoSmithKline, South Africa

Mr Magnifique Irakoze, Regional Coordinator for Africa, International Federation of Medical Students Associations, Rwanda

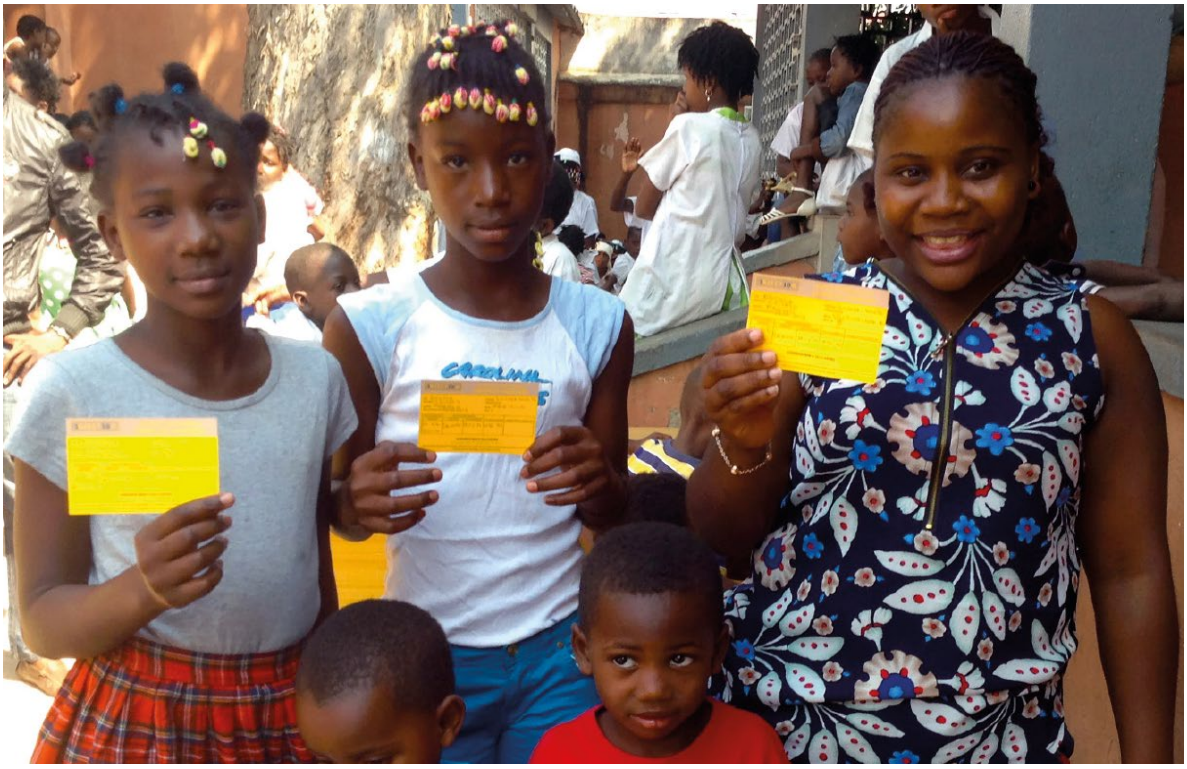

7:00 PM

Cocktail offered by the Government of Rwanda: Terrace Kigali Convention Centre

DAY 2
28 JUNE 2017

08:00 am – 09:00 am
Registration – Kigali Heights: 2nd floor Eastern Wing opposite the Kigali Convention Centre

Networking – Foyer 1A at the Kigali Convention Center

09:00 AM – 10:30 AM | Auditorium

Session 4: Making UHC work in Africa – How can the private sector contribute?

Strengthening existing partnerships and creating new connections will be key to attaining the goal of Universal Health Coverage – i.e. that all people obtain the health services they need without suffering financial hardship when paying for them. To achieve this goal, partnering with the private sector is fundamental. Finding new approaches to utilising partnerships has to come from all levels of government and beyond. Policymakers have to open the door for collaboration with the private sector, and partners have to create innovative solutions that can expand access to health services in new ways. The panel will discuss the role, importance and added value of private sector engagement in the context of Africa's efforts to achieve Universal Health Coverage.

Moderator

Ms Julie Gichuru, Founder and Chief Executive Officer at Arimus Media, Kenya

Panelists

Dr Solange Hakiba, Deputy Director General Benefits, Rwanda Social Security Board, Rwanda

Mr Jithu Jose, General Manager, Apollo Hospitals, India

Dr Belay Begashaw, Director General, Sustainable Development Goals Centre for Africa, Rwanda

Ms Adesimbo Ukiri MD/CEO, Avon Healthcare Limited, Nigeria

Dr Peter Okebukola, Associate Partner, McKinsey & Company, Nigeria

10:30 pm – 11:00 pm
Health break & networking – Foyer 1A

11:00 AM – 12:30 PM | Auditorium

Session 5: Old enemies (HIV, TB, Malaria), new threats (NCDs Urbanization, Climate change)

While facing a huge burden of communicable diseases, new threats such as non-communicable diseases and the impact of climate change are jeopardising the human, social and economic development of African populations. Equitable access to health care and prevention remains a distant goal in most countries. The demographic transition in the continent represents both an opportunity as well as a threat especially if suitable development options for healthcare and prevention are not adopted and implemented. The panel discussion will aim to inspire Africa led action to address challenges related to communicable, non-communicable diseases and environmental determinants of health.

Moderator

Mr Henry Bonsu, Journalist and Broadcaster, London, UK

Keynote speaker

Ambassador Macharia KAMAU, Kenya's Ambassador and Permanent Representative to the UN, USA

Panelists

Professor Jean-Claude Mbanya, Professor of Medicine and Endocrinology, University of Yaounde, Cameroon

Mr Pascal Nyamurinda, Mayor of Kigali, Rwanda

Hon. Dr Mohammad Anwar Husnoo, Minister of Health and Quality of Life, Mauritius

Professor Michael Marmot, Director Institute of Health, Equity, UK

12:30 pm – 2:00 pm
Lunch & networking –
Foyer 1A

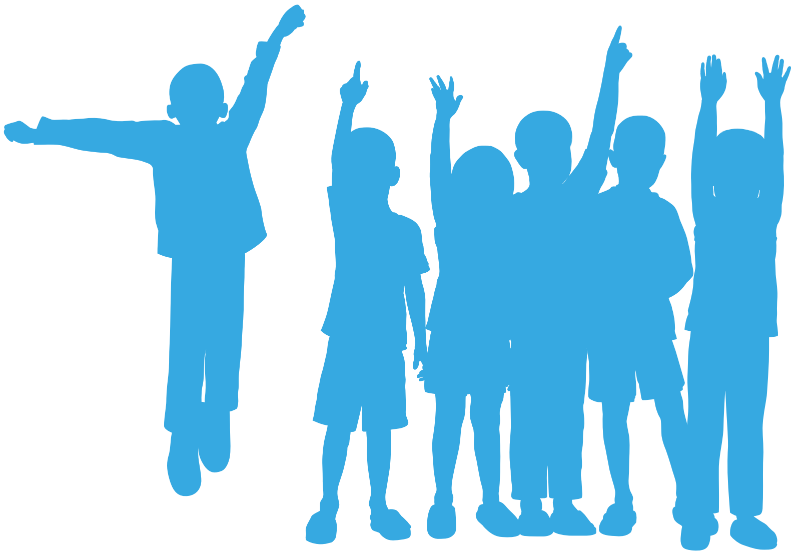

2:00 PM – 3:00 PM | Auditorium

Special event on Adolescent Health

The African continent is the only region in the world where the number of adolescents is predicted to increase over the next 50 years. The proportion of the world's adolescent and youth living in Africa is expected to rise from 18 % in 2012 to 28 % by 2040, while the shares for all other region will decline. Within this context, the health and development of Africa's adolescents will be key to achieving the Sustainable Development Goals.

Master of Ceremony

Dr Jeanine Condo, General Director , Rwanda Biomedical Center

Moderators

Ms Gogontlejang Phaladi, Devolpment Practitioner, AfriYAN

Mr Patrick Sewa Mwesigye, Country Director of AfriYAN, Executive Director of Uganda Youth and Adolescent Health Forum

Keynote speaker

Guest of Honor

Panelists

Ms Nadege Munyaburanga Uwase, Young girls, activist for adolescent health

Ms Francine Muyumba, President of the African Youth Union

Dr Felicitas Zawaira, Director Family and Reproductive Health, WHO

Mrs Julitta Onabanjo, UNFPA Regional Director

Dr. Valentina Baltag, Scientist,
WHO, Switzerland

Concluding remarks

Dr Matshidiso Moeti, WHO Regional Director for Africa

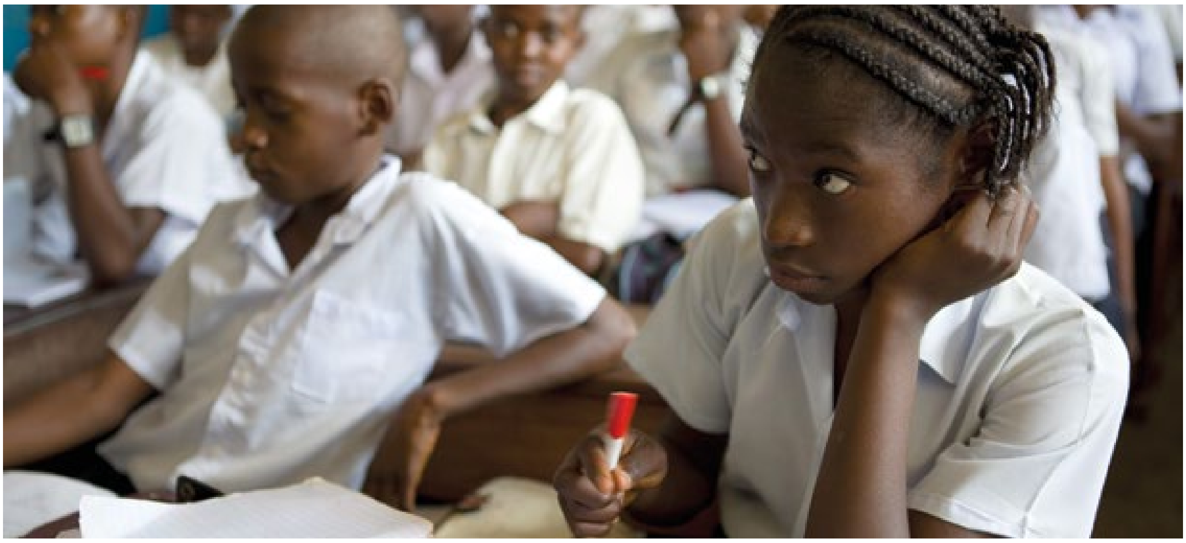

3:00 PM – 4:30 PM | Auditorium

Session 6: Putting people first – bringing better health to Africa's people

Countries in the African region have made commendable strides in health, however the newly adopted Sustainable Development Goals require greater efforts to ensure healthy lives and wellbeing for all. It is critical to improve the scope and mode of delivery of health services and also to address the existing inequities in order to have a meaningful impact on people's lives. The health sector interventions will need to be complemented by inter-sectorial action to effectively address the social, economic and environmental determinants of health. At the end it is important to identify ways countries can optimise the multiplicity of stakeholders and the different resources to build a resilient health system that will sustainably address the health and wellbeing of the people without leaving anyone behind.

Moderator

Ms Gogontlejang Phaladi, Founder and Executive Director, Gogontlejang Phaladi Pillar of Hope Organization, Botswana

Panelists

Professor Jean Claude Mbanya, Professor of Medicine and Endocrinology, Yaounde, Cameroon

Dr Timothy G. Evans, Senior Director, World Bank Group, USA

Dr Mary Amuyunzu Nyamongo, Founder Director and Technical Adviser, African Institute for Health and Development (AIHD), Kenya

Dr Solange Hakiba, Deputy Director General, Rwanda Social Security Board, Rwanda

Dr Bernice Dahn, Minister of Health, Liberia

4:30 pm – 5:00 pm

Health break & networking – Foyer 1A

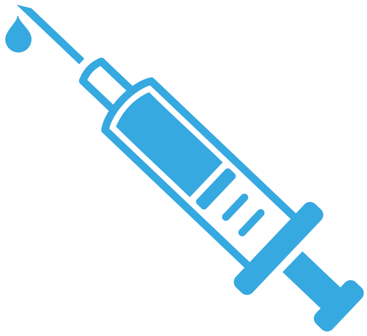

5:00 PM – 6:30 PM | Auditorium

Closing Ceremony

Master of Ceremony

Dr Jean Baptiste MAZARATI, Director, Bio-Medical Services, RBC

Presentation of the Call to Action

Dr Diane Gashumba, Minister of Health, Rwanda

Closing Remarks

Dr Matshidiso MOETI, WHO Regional Director for Africa

6.30 pm: End of the First WHO Africa Health Forum

Please note this is a preliminary programme and changes may occur.

World Health Organization – Regional Office for Africa
Cité du Djoué
P.O. Box 06 Brazzaville
Republic of Congo
0047 241 39100

Tel: + (242) 06 508 11 14
Fax: + (47 241) 39503
Email: AfricaHealthForum@WHO.int

www.afro.who.int

Download the Forum App on:

Apple Store   Google Store
